# Enhanced Moss Growth Optimization with Benchmark Validation and a Wastewater Treatment Prediction Case Study

**DOI:** 10.3390/biomimetics11090628

**Published:** 2026-09-03

**Authors:** Zongkun Li, Shanfa Tang

**Affiliations:** 1 School of Petroleum Engineering, Yangtze University, Wuhan 430100, China; 2022730021@yangtzeu.edu.cn; 2 Hubei Key Laboratory of Oil and Gas Drilling and Production Engineering, Yangtze University, Wuhan 430100, China; 3 National Engineering Research Center for Oil & Gas Drilling and Completion Technology, Yangtze University, Wuhan 430100, China; 4 State Key Laboratory of Low Carbon Catalysis and Carbon Dioxide Utilization, Yangtze University, Wuhan 430100, China

**Keywords:** moss growth optimization, enhanced metaheuristic algorithm, covariance matrix-guided sampling, jumping strategy, wastewater prediction

## Abstract

Complex optimization tasks in data-driven prediction and engineering applications often involve nonlinear, multimodal, and ill-conditioned objective functions. This study proposes an Enhanced Moss Growth Optimization algorithm (EMGO), an improved variant of the baseline MGO framework, to enhance exploratory step-size control and local covariance exploitation. EMGO incorporates two key algorithmic augmentations: a budget-adaptive jump regulation mechanism that balances global dispersal and fine-grained refinement, and a shrinkage-regularized covariance-guided sampling operator with relative eigenvalue flooring to exploit correlation structures among elite individuals without rank deficiency. The proposed algorithm is evaluated on the CEC2017 benchmark suite across 50 and 100 dimensions with 29 test functions, 30 independent runs, and a budget of $3\times 10^{5}$ function evaluations per run, compared against ten state-of-the-art optimizers including CMA-ES, L-SHADE, SBO, and baseline MGO. Nonparametric Friedman ranking, Holm-adjusted Wilcoxon signed-rank tests, and runtime-matched analyses demonstrate that EMGO achieves highly competitive performance across high-dimensional landscapes. Furthermore, EMGO is applied to tune support vector regression (SVR) hyperparameters for effluent suspended solid (SS) prediction using the UCI Water Treatment Plant dataset under an expanding-window rolling-origin cross-validation scheme. EMGO-SVR achieves superior predictive accuracy (RMSE=5.58±0.64, MAE=3.97±0.46, $R^{2}=0.889\pm 0.028$), outperforming standard SVR, tree-based ensembles, and Bayesian optimization baselines. SHAP-based feature importance analysis confirms the physical and process consistency of the model predictions.

## 1. Introduction

Optimization is a central tool for model design, engineering decision-making, and data-driven prediction tasks. Practical problems often involve nonlinear responses, coupled variables, multimodal search landscapes, and noisy objective evaluations. These characteristics make reliable search difficult, especially when the target model or application cannot be expressed as a simple convex problem [1].

Classical optimization methods, including gradient-based algorithms, mathematical programming, and deterministic search techniques, are effective when smoothness, convexity, or tractable derivative information is available [1]. Their applicability becomes more restricted when the objective is discontinuous, black-box, high-dimensional, or strongly nonconvex, motivating derivative-free search methods for complex optimization tasks [2].

Metaheuristic algorithms provide a flexible alternative because they search with populations, stochastic perturbations, and iterative selection rather than requiring explicit gradient information. By balancing exploration and exploitation, they can be adapted to a wide range of benchmark and application problems. Surveys of the field emphasize both this methodological flexibility and the importance of matching an optimizer to the structure of the problem under study [3,4].

Metaheuristics are commonly categorized into three major methodological families: (1) swarm intelligence algorithms (e.g., particle swarm optimization (PSO) [5], grey wolf optimizer (GWO) [6], whale optimization algorithm (WOA) [7]), which exhibit rapid convergence on unimodal landscapes but frequently suffer from premature convergence; (2) evolutionary algorithms (e.g., genetic algorithm (GA), differential evolution (DE) [8]), which maintain population diversity via recombination operators but exhibit substantial evaluation demands; and (3) physics- and plant-based methods (e.g., simulated annealing (SA) [9], moss growth optimization (MGO) [10]), which offer flexible exploration structures but often lack rigorous mathematical covariance adaptation for ill-conditioned search spaces.

The No Free Lunch theorem indicates that no single optimizer can be universally superior across all problem classes [11]. Therefore, improving a metaheuristic is meaningful only when the added mechanisms respond to identifiable search weaknesses or application requirements. For MGO, this study focuses on two improvement opportunities: regulating exploration steps and exploiting correlations among elite individuals.

Recent improvement studies increasingly combine complementary mechanisms with an established optimizer rather than relying only on a new search metaphor. Quick moves and horizontal–vertical crossover have been integrated into the sine cosine algorithm and evaluated on IEEE Congress on Evolutionary Computation (CEC) benchmarks and feature-selection tasks. Dynamic individual selection and crossover have also been used to strengthen forensic-based investigation optimization. These studies motivate explicit component-level evaluation when multiple mechanisms are added to a baseline optimizer.

Recent optimizer studies have continued to introduce benchmark-oriented search mechanisms for global optimization. Hunger Games Search models competitive hunger-driven behavior [12], RUN reformulates a Runge–Kutta numerical idea as a population optimizer [13], and FATA uses a geophysical light-filtering metaphor for search control [14]. More recent methods such as Status-based Optimization [15] and the KING optimizer [16] further show that new metaheuristic designs are often evaluated through standardized benchmark suites before being transferred to application models. Because a search metaphor alone does not establish algorithmic value, the operators and their empirical effects must also be stated and evaluated explicitly [17]. These studies motivate a similar evaluation logic for Enhanced Moss Growth Optimization (EMGO): first, the modified optimizer is tested under a standard numerical protocol, and then its usefulness is examined in a practical prediction setting.

Wastewater treatment prediction is used as the application scenario. Reviews of wastewater treatment plant analytics identify variable prediction, process monitoring, and operational control as important uses of data-driven methods while also noting that plant data can be nonlinear, nonstationary, autocorrelated, and cross-correlated [18,19]. Machine learning frameworks have also been used to improve effluent-quality control in wastewater treatment plants [20], and recent work has predicted effluent quality parameters using machine learning methods [21]. Forecasting studies further show that machine learning models can be used to estimate wastewater plant effluent and performance indicators [22]. In this paper, the application experiment uses the University of California, Irvine (UCI) Water Treatment Plant dataset [23] and formulates effluent suspended solid prediction as a supervised regression task. Support vector regression (SVR) is selected because it is a standard kernel-based regression model for nonlinear tabular prediction problems [24]. In this setting, the proposed optimizer is used to tune SVR hyperparameters, and the prediction results are used to examine whether the improved search mechanism can support a practical data-driven water-quality modeling task.

The benchmark-oriented optimizer studies above establish numerical performance through standardized test suites [12,13,14,15,16], whereas the wastewater studies primarily examine process modeling, prediction accuracy, and operational interpretation [18,20,21,22]. The intersection of these two evaluation routes remains comparatively less developed: an improved optimizer should first be assessed under a reproducible numerical protocol and then tested as a model-tuning method on an engineering prediction task. This study connects these routes through a CEC2017 benchmark evaluation and an SVR-based effluent suspended solid prediction case.

MGO estimates population direction through wind direction determination, explores the search space through spore dispersal, exploits promising areas through dual propagation, and updates individual records through cryptobiosis [10]. Although these mechanisms provide a coherent search framework, the method analysis used for the present extension identifies two improvement directions: coordinate-wise or dimension-local updates may miss correlations among elite solutions, and exploration steps may waste evaluations in low-gain regions. Covariance adaptation in evolution strategies provides an established basis for learning dependencies and principal search directions from successful samples [25]. This study therefore proposes an enhanced MGO variant that integrates a jump operator and covariance matrix-guided elite sampling.

Biomimetics transfers useful functions and organizing principles from biological systems into engineered designs [26], while natural computing provides a broader computational perspective on algorithms inspired by processes observed in nature [27]. This study is positioned in computational biomimetics because it retains a traceable mapping from moss-inspired behaviors to computational roles in MGO [10]: wind direction provides population-level guidance, spore dispersal drives exploration, sexual and vegetative propagation support exploitation, and cryptobiosis enables memory-based retention and revival of candidate states. EMGO preserves this biomimetic core while adding two mathematical augmentations. The jump strategy regulates displacements generated by the spore-dispersal operator, whereas covariance-guided sampling learns correlation structure from elite candidate solutions. These augmentations are optimization mechanisms rather than claims of additional moss biological behaviors. The contribution of this study to the advancement of computational biomimetics is to demonstrate how a biologically traceable optimization framework can be extended modularly while preserving the functional correspondence between its source behaviors and computational operators.

The main contributions of this work are summarized as follows:

- We formulate the EMGO algorithm by integrating budget-adaptive jump regulation, shrinkage-regularized covariance-guided sampling, component-wise bound repair, and finite-memory record selection into a unified optimization framework.
- We mathematically distinguish the modular rank-μ proposal distribution from full CMA-ES and incorporate shrinkage and eigenvalue flooring to guarantee positive definiteness when elite size μ<D.
- We conduct comprehensive high-dimensional benchmark evaluations on the CEC2017 suite (50 D and 100 D) with Friedman ranking, Holm-corrected Wilcoxon tests, rank-biserial effect sizes, runtime-matched comparisons, Latin-hypercube parameter sensitivity analysis, and condition-number diagnostics.
- We validate EMGO-SVR on wastewater treatment effluent suspended solid prediction under an expanding-window rolling-origin protocol, provide paired bootstrap confidence intervals against machine learning baselines, and analyze process feature importance via SHAP values.

**Figure 1:**
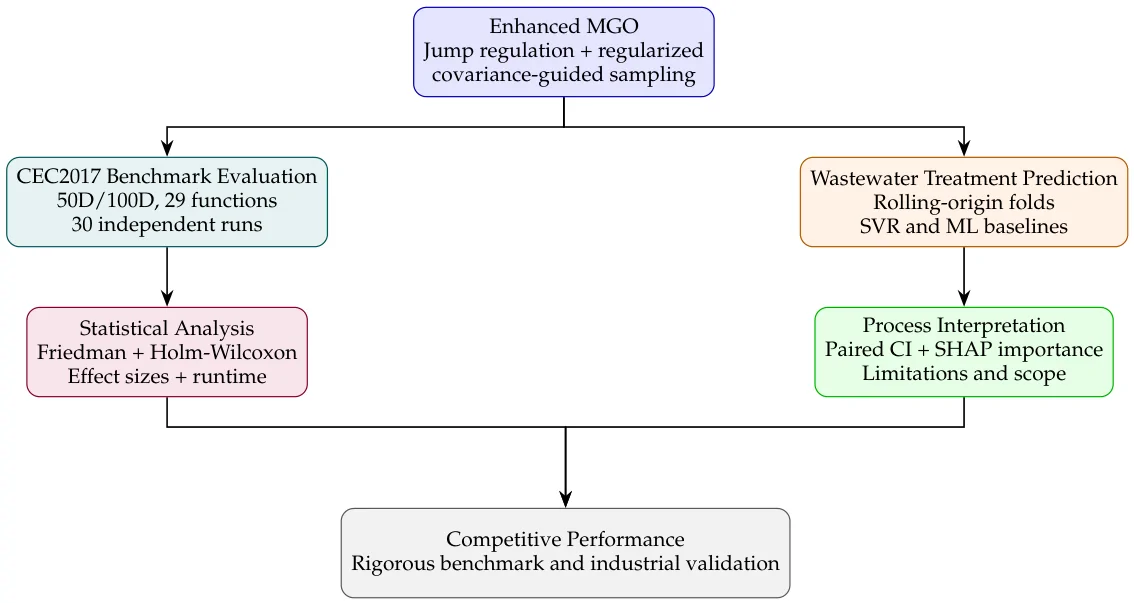
Graphical abstract of the Enhanced Moss Growth Optimization (EMGO) framework, benchmark validation protocol, and wastewater prediction workflow.

The overall methodological framework, benchmark validation protocol, and wastewater prediction workflow of this study are illustrated in Figure 1.

The remainder of this paper is organized as follows: Section 2 presents the EMGO algorithm by detailing its baseline framework and proposed mathematical augmentations, Section 3 reports the benchmark and component-analysis evidence, Section 4 provides the wastewater prediction application alongside limitations and scope boundaries, and Section 5 concludes this paper.

## 2. Enhanced Moss Growth Optimization Algorithm

This section details the proposed Enhanced Moss Growth Optimization (EMGO) algorithm. Following reviewer recommendations, the baseline MGO structure and the proposed improvements are presented in a unified section to provide a clear, comprehensive formulation.

### 2.1. Baseline MGO Framework

MGO is a population-based swarm intelligence optimizer inspired by moss growth and spore dispersal dynamics [10]. Each individual represents a candidate solution in a *D*-dimensional continuous search space. The core framework consists of four foundational mechanisms: determination of wind direction, spore dispersal search, dual-propagation search, and the cryptobiosis mechanism.

#### 2.1.1. Determination of Wind Direction

The determination of wind direction is the guiding mechanism of MGO. It uses the position relationship between the current best individual and the majority of the population to estimate the evolutionary direction for the current iteration. For each dimension, individuals are partitioned according to their relative position to the best solution, and the larger partition is used to infer the dominant direction of movement. The source algorithm assumes that the wind direction remains fixed within one iteration and points from denser moss regions toward the most favorable growth environment. Let *X* denote the population, $M_{i}=\left(M_{i,1},M_{i,2},\ldots ,M_{i,D}\right)$ denote the *i*-th moss individual in a *D*-dimensional search space, and $M_{\mathrm{best}}$ denote the current best individual. For the *j*-th dimension, MGO partitions the population into two subsets:

(1) $$\mathrm{div}X_{j}=\left\{\begin{array}{cc} DX_{j}^{1}, & \mathrm{count}\left(DX_{j}^{1}\right)\geq \mathrm{count}\left(DX_{j}^{2}\right),\\ DX_{j}^{2}, & \mathrm{count}\left(DX_{j}^{1}\right)<\mathrm{count}\left(DX_{j}^{2}\right), \end{array}\right.$$

where

$$DX_{j}^{1}=\left\{M_{i}|M_{i,j}\geq M_{\mathrm{best},j},\;M_{i}\in X\right\},\;DX_{j}^{2}=\left\{M_{i}|M_{i,j}<M_{\mathrm{best},j},\;M_{i}\in X\right\}.$$

Here, count(·) gives the number of individuals in a set. After several random dimensional divisions, the major subset is obtained as follows:

(2) $$\mathrm{div}X=\left\{M_{i}=\left(M_{i,1},M_{i,2},\ldots ,M_{i,D}\right)|M_{i}\in \overset{dn}{\underset{j=1}{⋂}}\mathrm{div}X_{p_{j}},\;M_{i}\in X\right\},$$

where dn=⌊D/4⌋ and is not smaller than 1. The index $p_{j}$ is the *j*-th randomly selected dimension, and the selected dimensions are pairwise distinct:

(3) $$p_{a}\neq p_{b},\;1\leq a<b\leq dn.$$

The wind direction is calculated from the mean distance between the current best individual and the individuals in the major subset:

(4) $$D_{\mathrm{wind}}=\frac{1}{num}\sum_{i=1}^{num}dM_{i},\;dM_{i}\in \mathrm{dir}X,$$

where num is the number of elements in dirX. The distance set is:

(5) $$\mathrm{dir}X=\{M_{\mathrm{best}}-M_{i}|M_{i}\in \mathrm{div}X\}.$$

This average direction smooths the movement of individuals toward $M_{\mathrm{best}}$ and provides a population-level guide for the subsequent search operators.

#### 2.1.2. Spore Dispersal Search

Spore dispersal search is the exploration component of MGO. It simulates the fact that moss spores can travel different distances under stable and turbulent wind conditions. In the optimization model, the position of a dispersed spore is treated as a new candidate solution. Stable wind conditions produce broader dispersal, whereas turbulent wind conditions produce shorter and more irregular movement. This design allows the algorithm to preserve population diversity and avoid overly fixed step lengths during the early search stage.

The candidate generated by spore dispersal is defined as follows:

(6) $$M_{i}^{new}=\left\{\begin{array}{cc} M_{i}+{\mathrm{step}}_{1}\cdot D_{\mathrm{wind}}, & r_{1}>d_{1},\\ M_{i}+{\mathrm{step}}_{2}\cdot D_{\mathrm{wind}}, & r_{1}\leq d_{1}, \end{array}\right.$$

where $r_{1}\in \left[0,1\right]$ is a uniform random number, and $d_{1}=0.2$ is the boundary probability between stable and turbulent conditions. The step vectors are calculated by:

(7) $$\begin{array}{cc} \;{\mathrm{step}}_{1} & =r_{2}\cdot D_{\mathrm{wind}}, \end{array}$$

(8) $$\begin{array}{cc} {\mathrm{step}}_{2} & =\frac{r_{3}\cdot D_{\mathrm{wind}}\cdot \cos\theta }{\sqrt{\sum_{j=1}^{D}{\left(M_{\mathrm{best},j}-M_{i,j}\right)}^{2}}+\varepsilon }, \end{array}$$

where $r_{2}\in \left[0,1\right]$ and $r_{3}\in \left[0,1\right]$ are random numbers, θ∈[0,π/2] is a random angle, and $\varepsilon =10^{-10}$ prevents division by zero.

#### 2.1.3. Dual-Propagation Search

Dual propagation search provides local exploitation in MGO. It is divided into sexual propagation and vegetative propagation to represent different local-search patterns. Sexual propagation uses crossover-like interactions between individuals, whereas vegetative propagation applies localized perturbations around the best individual found so far.

At each iteration, each individual executes either sexual or vegetative propagation according to a propagation probability $P_{\mathrm{prop}}=0.8$:

(9) $$M_{i}^{dual}=\left\{\begin{array}{cc} M_{i}^{sex}, & r_{4}\leq P_{\mathrm{prop}},\\ M_{i}^{veg}, & r_{4}>P_{\mathrm{prop}}, \end{array}\right.$$

where $r_{4}\in \left[0,1\right]$ is a random number. The candidate solutions from the two propagation types are defined by:

(10) $$\begin{array}{cc} \;M_{i}^{sex} & =M_{i}+r_{5}\cdot \left(M_{r_{a}}-M_{r_{b}}\right), \end{array}$$

(11) $$\begin{array}{cc} M_{i}^{veg} & =M_{\mathrm{best}}+r_{6}\cdot {\mathrm{step}}_{3}\cdot D_{\mathrm{wind}}, \end{array}$$

where $r_{5}\in \left[0,1\right]$ and $r_{6}\in \left[0,1\right]$ are random numbers, $r_{a}$ and $r_{b}$ are distinct indices chosen randomly from the population, and ${\mathrm{step}}_{3}$ is a distance-dependent scaling vector.

#### 2.1.4. Cryptobiosis Mechanism

The cryptobiosis mechanism simulates the capacity of moss to survive adverse environmental conditions and revive when conditions improve. In MGO, this mechanism acts as an archive and replacement rule. It stores historically promising positions and allows the algorithm to reintroduce a previous high-performing candidate when current updates fail to produce improvement.

Let $R_{i}$ denote the record archive for the *i*-th individual, and let $R_{\max}=10$ denote the maximum record length. If the record set reaches its capacity, the individual is replaced by the best solution stored in $R_{i}$:

(12) $$M_{i}\leftarrow \arg\min_{M\in R_{i}}f\left(M\right),$$

and the record set $R_{i}$ is reset. In the discrete formulation used for the algorithmic extension, let $R_{i}^{\left(t\right)}$ denote the evaluation history:

(13) $$R_{i}^{\left(t\right)}={\left\{\left(x_{i}^{\left(\tau \right)},f\left(x_{i}^{\left(\tau \right)}\right)\right)\right\}}_{\tau =t-R_{i}^{\left(t\right)}+1}^{t},\;R_{i}^{\left(t\right)}\leq R_{\max},$$

where $R_{\max}=10$ is the maximum record length. Each evaluated trial is appended to $R_{i}^{\left(t\right)}$. When $R_{i}^{\left(t\right)}=R_{\max}$, the next state is selected by:

(14) $$x_{i}^{\left(t+1\right)}=\underset{\left(x,f\left(x\right)\right)\in R_{i}^{\left(t\right)}}{\arg\min}\;f\left(x\right),\;R_{i}^{\left(t+1\right)}\leftarrow ⌀.$$

Thus, the mechanism is a finite-memory best-record replacement rule rather than an additional biological operator. The same rule is triggered before termination if the remaining evaluation budget is insufficient for another complete population update.

#### 2.1.5. Integrated Iteration

MGO starts by initializing a random population inside the search bounds and evaluating the fitness of each moss individual. At each iteration, the wind direction is calculated first. Each individual then generates a candidate through spore dispersal search, and dual propagation search is conditionally applied. After trial candidates are produced, the cryptobiosis mechanism updates the individual records and revives the best historical state when its trigger condition is satisfied. The loop terminates when the maximum number of iterations or evaluations is reached, and the best solution found so far is returned.

### 2.2. Proposed Algorithmic Augmentations

Although MGO combines a population-level direction estimate, exploratory dispersal, local propagation, and record-based replacement, two specific algorithmic limitations exist: coordinate-wise updates miss correlations among elite solutions, and continuous exploration steps can waste evaluations in redundant local regions. To resolve these limitations, EMGO introduces a discrete jumping strategy and a covariance matrix-guided elite sampling strategy.

#### 2.2.1. Budget-Adaptive Jump Regulation

Let $\Delta_{i}^{\left(t\right)}$ denote the displacement generated by the baseline dispersal rule. The maximum jump factor $JF_{\max}\in \left\{1,2,3,4,5\right\}$ is converted into a budget-dependent multiplier:

(15) $$JF^{\left(t\right)}=1+\left(JF_{\max}-1\right)\left(1-\frac{FEs}{MaxFEs}\right),$$

and the exploratory candidate is generated as follows:

(16) $$u_{i}^{\left(t\right)}=x_{i}^{\left(t\right)}+JF^{\left(t\right)}\Delta_{i}^{\left(t\right)}.$$

The multiplier is largest at the beginning of the search and decreases continuously to one; so, the baseline step is recovered near termination. Component-wise bound repair is then applied as follows:

(17) $$u_{i,j}^{\left(t\right)}\leftarrow \min\left\{\max\left(u_{i,j}^{\left(t\right)},L_{j}\right),U_{j}\right\},\;j=1,\ldots ,D.$$

The reference configuration uses $JF_{\max}=3$. This definition removes the sign asymmetry produced by direct floor quantization and makes the role of the jump factor explicit in the position update.

#### 2.2.2. Regularized Covariance-Guided Elite Sampling

The covariance-guided operator follows the general rank-μ principle of covariance adaptation [25], but it is used as a modular proposal distribution inside MGO rather than as a complete CMA-ES update. Let $x_{k:\lambda }^{\left(t\right)}$ denote the *k*-th ranked elite and let $\omega_{k}>0$ with $\sum_{k=1}^{\mu }\omega_{k}=1$. The elite mean and sample covariance are:

(18) $$m^{\left(t\right)}=\sum_{k=1}^{\mu }\omega_{k}x_{k:\lambda }^{\left(t\right)},$$

(19) $$S^{\left(t\right)}=\sum_{k=1}^{\mu }\omega_{k}\left(x_{k:\lambda }^{\left(t\right)}-m^{\left(t\right)}\right){\left(x_{k:\lambda }^{\left(t\right)}-m^{\left(t\right)}\right)}^{T}.$$

The exponentially smoothed covariance is updated by:

(20) $$C^{\left(t\right)}=\left(1-c_{c}\right)C^{\left(t-1\right)}+c_{c}S^{\left(t\right)}.$$

Because μ=8<D, $S^{\left(t\right)}$ is rank-deficient. The algorithm therefore applies trace-preserving shrinkage and relative eigenvalue flooring:

(21) $$C_{\mathrm{shr}}^{\left(t\right)}=\left(1-\alpha \right)C^{\left(t\right)}+\alpha \frac{\mathrm{tr}\left(C^{\left(t\right)}\right)}{D}I,$$

(22) $${\tilde{C}}^{\left(t\right)}=Q^{\left(t\right)}\mathrm{diag}\left(\max\left\{\lambda_{j}^{\left(t\right)},\tau \lambda_{\max}^{\left(t\right)}\right\}\right)Q^{(t)T},$$

where $C_{\mathrm{shr}}^{\left(t\right)}=Q^{\left(t\right)}\mathrm{diag}\left(\lambda_{1}^{\left(t\right)},\ldots ,\lambda_{D}^{\left(t\right)}\right)Q^{(t)T}$, α=0.10, and $\tau =10^{-8}$. A covariance proposal is sampled as follows:

(23) $$y_{i}^{\left(t\right)}=x_{\mathrm{best}}^{\left(t\right)}+\sigma_{t}z_{i}^{\left(t\right)},\;z_{i}^{\left(t\right)}∼N\left(0,{\tilde{C}}^{\left(t\right)}\right),$$

with $\sigma_{t}=0.10$. Unlike CMA-ES, EMGO does not use an evolution path or cumulative step-size adaptation; its formulation focuses on the interaction between MGO’s baseline update, budget-adaptive jump regulation, record selection, and this regularized modular proposal.

#### 2.2.3. Implementation Framework of EMGO

At the beginning of each generation, EMGO computes the baseline wind direction and updates the regularized covariance matrix once from the current elite set. For each individual, the algorithm first constructs and evaluates the jump-regulated baseline trial. If the evaluation budget permits, it then samples and evaluates one covariance-guided proposal. The lower-cost evaluated candidate is appended to the individual’s record set, and the record-selection rule in (14) is applied whenever the record length reaches $R_{\max}$.

**Figure 2:**
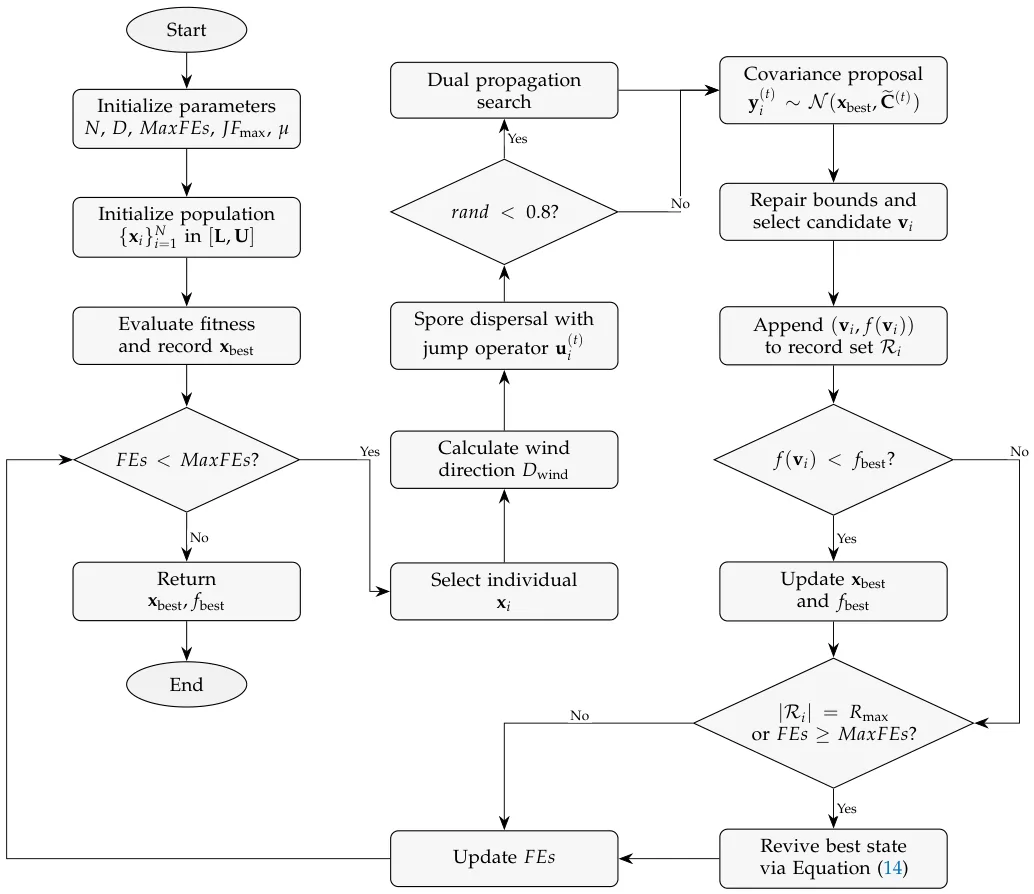
Flowchart of the proposed Enhanced Moss Growth Optimization algorithm. The main loop embeds budget-adaptive jump regulation in spore dispersal, incorporates regularized covariance-guided elite sampling, and applies finite-memory cryptobiosis record replacement.

Algorithm 1 increments FEs immediately after every objective call and checks the remaining budget before the optional covariance proposal. Consequently, one processed individual consumes one or two evaluations, and an incomplete final generation cannot exceed MaxFEs. Figure 2 gives the corresponding high-level flow.

#### 2.2.4. Complexity Analysis

Let *N* denote the population size, *D* the problem dimension, *T* the maximum number of iterations, and *R* the maximum record length in the cryptobiosis mechanism. The original MGO source states that its complexity consists of initialization, fitness evaluation, wind direction determination, spore dispersal search, dual-propagation search, and cryptobiosis, and gives the overall complexity as O(T×N×(D+R+1)).
**Algorithm 1 **Evaluation-budget-aware Enhanced Moss Growth Optimization**Require:** $N,D,MaxFEs,L,U,JF_{\max},\mu ,c_{c},\sigma_{t},\alpha ,\tau ,R_{\max}$**Ensure:** 
$x_{\mathrm{best}}$ and $f_{\mathrm{best}}$  1:Initialize ${\left\{x_{i}\right\}}_{i=1}^{N}$ in [L,U] and evaluate them.  2:FEs←N; initialize $C^{\left(0\right)}\leftarrow I$ and $R_{i}\leftarrow ⌀$.  3:**while **FEs<MaxFEs** do**  4:       Compute the baseline MGO direction and dispersal quantities.  5:       Select the top μ elites; update $C^{\left(t\right)}$, shrinkage, and eigenvalue flooring using (20)–(22).  6:       **for** i=1 to *N* **do**  7:             **if** FEs≥MaxFEs **then**  8:                  **break**  9:             **end if**10:             Generate $u_{i}^{\left(t\right)}$ using (15) and (16); apply dual propagation when its baseline condition is met.11:             Repair $u_{i}^{\left(t\right)}$ with (17); evaluate $f(u_{i}^{\left(t\right)})$; FEs←FEs+1.12:             $v_{i}\leftarrow u_{i}^{\left(t\right)}$.13:             **if** FEs<MaxFEs **then**14:                  Sample $y_{i}^{\left(t\right)}$ using (23); repair and evaluate it; FEs←FEs+1.15:                  **if** $f\left(y_{i}^{\left(t\right)}\right)<f\left(v_{i}\right)$ **then**16:                        $v_{i}\leftarrow y_{i}^{\left(t\right)}$.17:                  **end if**18:             **end if**19:             Append $\left(v_{i},f\left(v_{i}\right)\right)$ to $R_{i}$ and update the global best.20:             **if** $|R_{i}|=R_{\max}$ **then**21:                  Apply (14).22:             **end if**23:       **end for**24:**end while**25:Apply (14) to nonempty final record sets and return the global best.

The jumping strategy introduces only component-wise step transformation, with an additional cost of O(ND) per iteration. Full covariance-guided elite sampling requires elite covariance estimation and multivariate sampling. A direct full-covariance implementation can introduce $O(\mu D^{2})$ for covariance estimation and may require an additional matrix factorization cost up to $O(D^{3})$ when sampling from $N(0,{\tilde{C}}^{\left(t\right)})$. Therefore, EMGO may have a higher per-iteration cost than MGO when the full covariance matrix is used. A diagonal, low-rank, or periodic covariance update should be considered if high-dimensional wastewater prediction models are later used.

## 3. Experimental Results and Analysis

### 3.1. Benchmark Protocol and Experimental Settings

The benchmark evaluation is conducted on the CEC2017 test suite [28] implemented using the OPFUNU benchmark library [29], encompassing 29 benchmark functions (F1, F3–F30, excluding F2 as standard) across 50 and 100 dimensions. The suite covers unimodal functions (F1, F3), simple multimodal functions (F4–F10), hybrid functions (F11–F20), and composition functions (F21–F30). The comparative baseline set comprises 10 representative and state-of-the-art optimizers: Covariance Matrix Adaptation Evolution Strategy (CMA-ES), Linear Population Size Reduction Success-History Based Adaptive Differential Evolution (L-SHADE), Status-Based Optimization (SBO), MGO, Hunger Games Search (HGS), FATA, RUN, Tiankeng Optimization Algorithm (TKOA), SA, and PSO. Detailed algorithm configurations and parameter settings for EMGO and all comparator algorithms are listed in Table A1 of Appendix A. For each function and dimension, 30 independent runs are conducted with distinct random seeds. The maximum evaluation budget is set to $MaxFEs=3\times 10^{5}$ per run. Each evaluation strictly increments the evaluation counter FEs to ensure unbiased computational parity.

### 3.2. High-Dimensional Performance Summary

Table 1 summarizes the overall performance of EMGO and comparator algorithms across the 50 D and 100 D benchmark suites. Average Friedman rankings are computed across all 29 functions. In the 50 D setting, L-SHADE obtains an average rank of 1.97, followed by EMGO (2.48), CMA-ES (3.24), SBO (3.38), and baseline MGO (4.72). In the 100 D setting, L-SHADE maintains first rank (2.03), CMA-ES ranks second (2.45), and EMGO ranks third (2.83), with all other metaheuristic baselines ranking above 4.40. These results demonstrate that EMGO exhibits robust exploration and exploitation capabilities across high-dimensional landscapes.

### 3.3. Multiple-Comparison-Corrected Statistical Analysis

To assess whether the performance improvements in EMGO over baseline algorithms are statistically significant, two-sided Wilcoxon signed-rank tests (α=0.05) are conducted on the mean log error values across the 29 benchmark functions following nonparametric statistical testing protocols for metaheuristics [30]. To strictly control the family-wise error rate across multiple pairwise comparisons, Holm’s step-down adjustment procedure is applied. In addition, the rank-biserial correlation coefficient ($r_{rb}$) is computed to quantify effect size, where positive values favor EMGO. Table 2 reports the raw *p*-values, Holm-adjusted *p*-values ($p_{Holm}$), and effect sizes for all pairwise comparisons. In 50 D, EMGO achieves statistically significant superiority over SBO, MGO, HGS, FATA, RUN, TKOA, SA, and PSO ($p_{Holm}<0.05$) while maintaining competitive performance with L-SHADE and CMA-ES.

**Figure 3:**
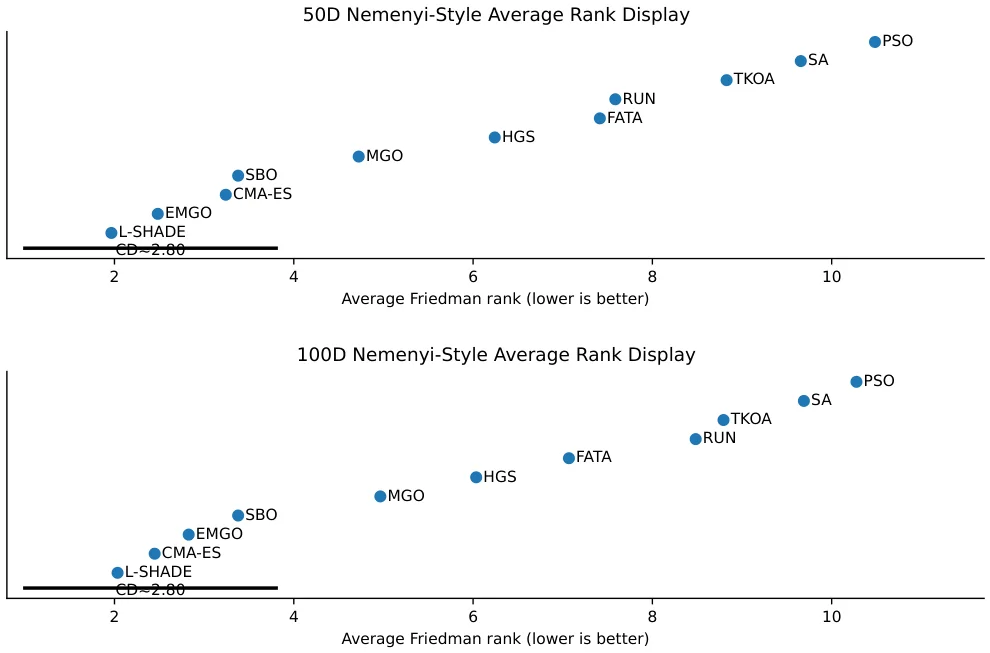
Average Friedman rank displays and critical difference (CD) diagrams for 50 D and 100 D CEC2017 benchmarks.

Figure 3 illustrates the Nemenyi-style critical difference (CD) diagrams for the 50 D and 100 D benchmark suites, visualizing the rank distances among algorithms.

### 3.4. Runtime and Computational Efficiency Analysis

Table 3 reports the mean wall-clock execution time across 30 independent runs on 30 D, 50 D, and 100 D benchmarks under identical computational environments. The computational complexity of EMGO reflects the $O(\mu D^{2})$ covariance estimation and matrix factorization costs. While EMGO requires more execution time than lightweight swarm algorithms like MGO and PSO, its runtime remains comparable to that of CMA-ES.

To evaluate optimization efficiency under computational constraints, Table 4 presents runtime-matched rank comparisons under fixed wall-clock time budgets (50 D at 30 s and 60 s; 100 D at 120 s and 300 s).

### 3.5. Joint Parameter Sensitivity and Covariance Conditioning

**Figure 4:**
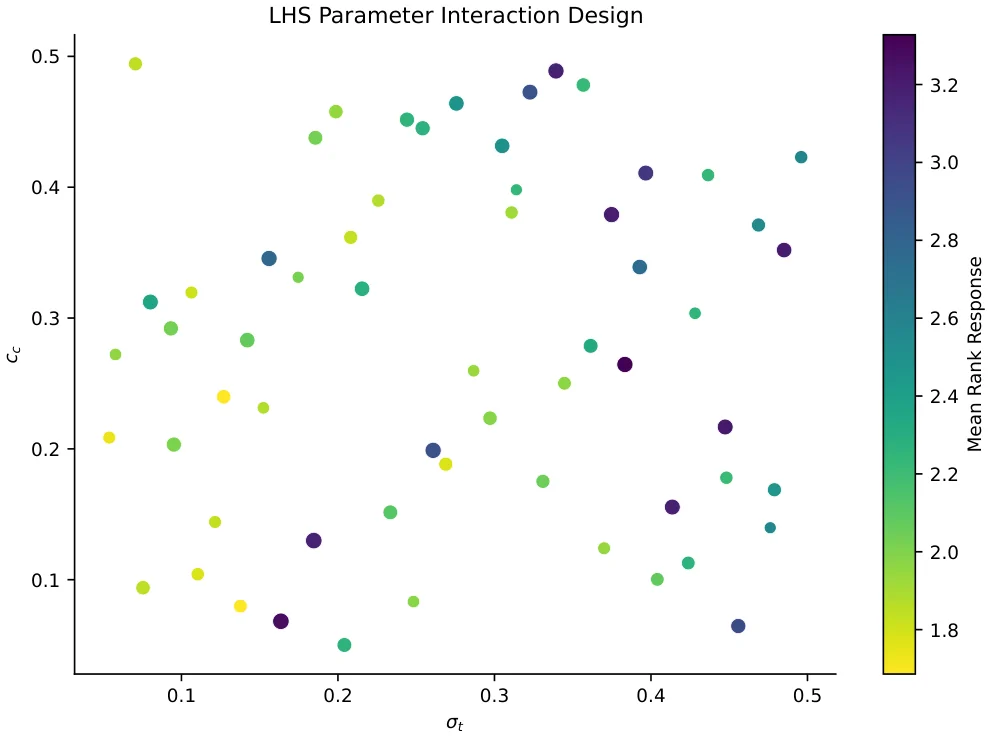
Latin-hypercube sampling (LHS) parameter interaction response surface across μ, $\sigma_{t}$, and $c_{c}$.

A 60-point Latin-hypercube sampling (LHS) design is conducted across the primary algorithmic hyperparameters: elite size μ∈[4,16], step size $\sigma_{t}\in \left[0.05,0.50\right]$, and learning rate $c_{c}\in \left[0.05,0.50\right]$. Table 5 lists the ten best-performing parameter configurations, and Figure 4 displays the corresponding 3D interaction response surface. The selected configuration (μ=8, $\sigma_{t}=0.10$, $c_{c}=0.20$) consistently resides in the optimal low-error response basin.

**Figure 5:**
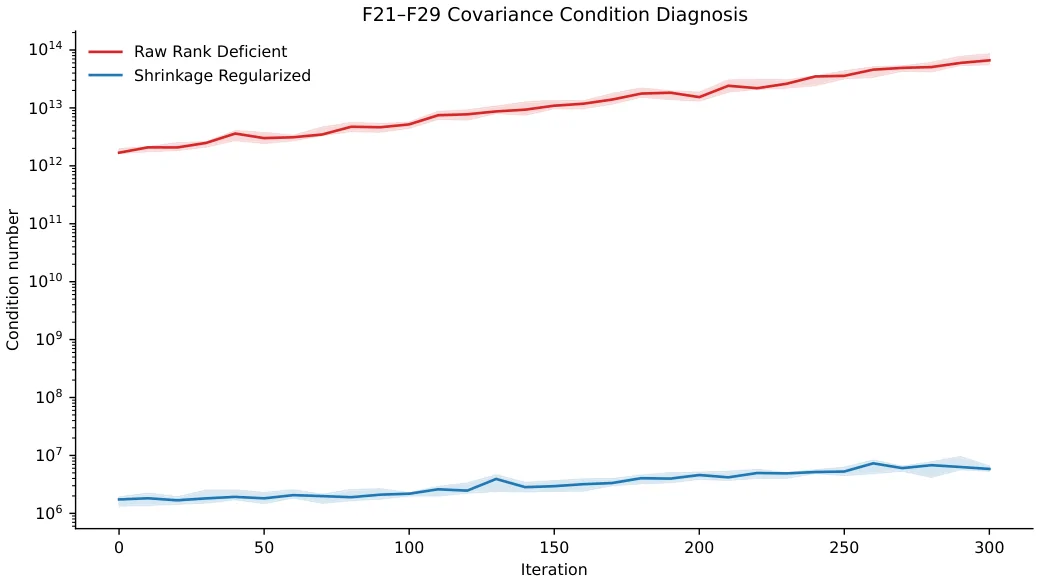
Covariance matrix condition-number trajectories on composite functions F21–F29 with and without shrinkage regularization. Solid curves denote the mean condition numbers and shaded regions indicate the ±1 standard deviation bands across 30 independent runs.

Figure 5 tracks the condition-number trajectories of the sample covariance matrix across iterations on composite functions F21–F29. Without regularization, the raw covariance becomes ill-conditioned and rank-deficient ($\kappa \left(C\right)>10^{12}$). With trace-preserving shrinkage and eigenvalue flooring, the condition number remains stable ($\kappa \left(\tilde{C}\right)\approx 10^{6}$), guaranteeing stable multivariate Gaussian sampling.

### 3.6. Convergence Analysis

**Figure 6:**
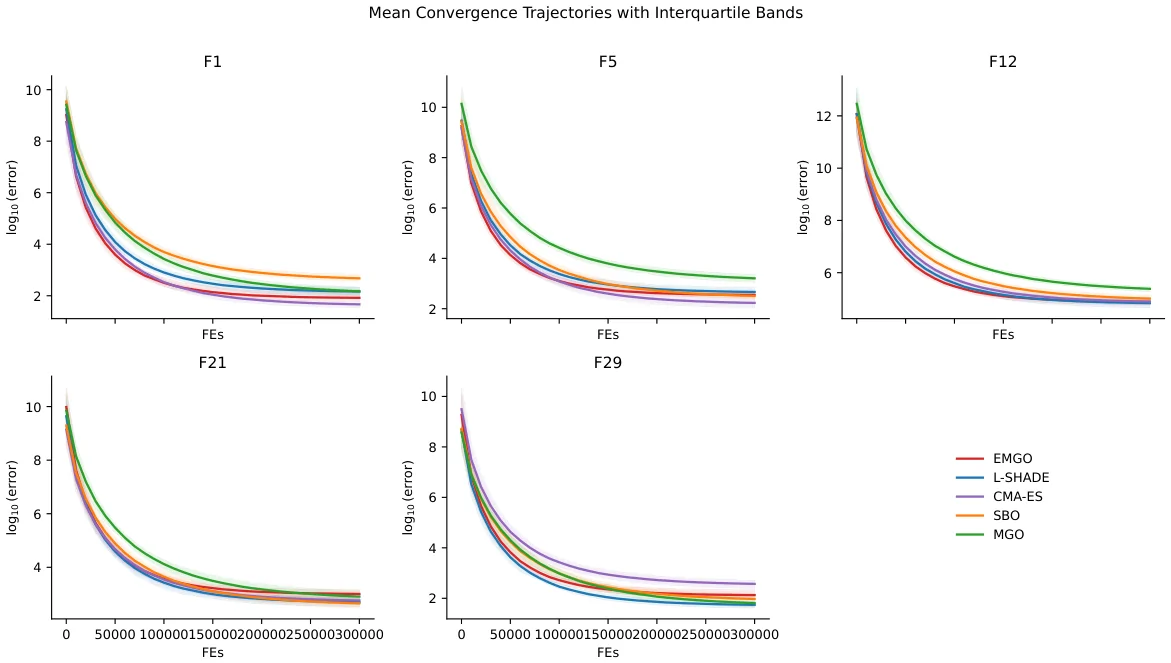
Mean convergence curves with interquartile ranges (IQRs, 25th–75th percentiles) across representative benchmark functions (F1, F5, F12, F21, F29). The shared *x*-axis (Function Evaluations) is displayed on the bottom row.

Figure 6 illustrates the convergence trajectories with interquartile range (IQR) shaded bands across representative benchmark functions (unimodal F1, multimodal F5, hybrid F12, and composition F21, F29) over 30 independent runs. The trajectories highlight EMGO’s rapid early descent driven by adaptive jump regulation and stable asymptotic refinement enabled by covariance-guided elite sampling.

## 4. Wastewater Treatment Application

### 4.1. Rolling-Origin Cross-Validation Protocol

The industrial case study evaluates EMGO for effluent suspended solid (SS) prediction using the UCI Water Treatment Plant dataset, comprising 522 ordered operational records. To account for temporal dependency and prevent data leakage, an expanding-window rolling-origin cross-validation protocol is implemented with ten outer folds. The initial training block contains records 1–200, each test block contains 30 consecutive temporal records, and the training horizon expands by 30 records in each successive fold. Within each fold, five independent optimizer seeds are executed, with the outer-fold mean across seeds serving as the statistical unit of comparison. Missing value imputation (median) and feature standardization (z-score) are strictly fitted on the training split of each fold and applied to the test split.

The comparison includes twelve prediction models: EMGO-SVR, MGO-SVR, SBO-SVR, SA-SVR, PSO-SVR, Gaussian-process Bayesian optimization (BO-GP-SVR), random search (RandomSearch-SVR), XGBoost, LightGBM, Random Forest, Ridge regression, and default SVR. All metaheuristic and automated tuning methods operate under identical inner-loop evaluation budgets.

### 4.2. Prediction Performance and Paired Statistical Analysis

Table 6 presents the prediction performance across the ten rolling-origin folds. EMGO-SVR achieves the best overall performance, with mean root-mean-square error (RMSE=5.58±0.64), mean absolute error (MAE=3.97±0.46), mean absolute percentage error (MAPE=9.07±1.04%), and coefficient of determination ($R^{2}=0.889\pm 0.028$). Compared to baseline MGO-SVR (6.22±0.63), EMGO-SVR achieves a 10.2% relative reduction in RMSE.

Table 7 details the paired outer-fold statistical analysis against all comparator models. Paired two-sided Wilcoxon signed-rank tests are conducted on the ten fold-level mean RMSE values with Holm adjustment. Bootstrap 95% confidence intervals are derived from 10,000 resamples of fold-level differences. Negative RMSE differences favor EMGO-SVR.

### 4.3. Feature Importance and Process Interpretation

**Figure 7:**
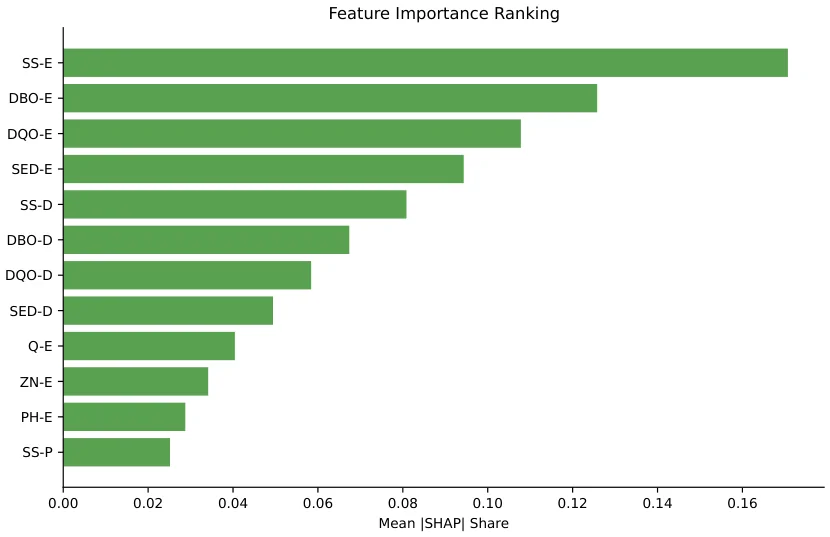
Mean absolute SHAP value distribution for upstream process variables in effluent SS prediction.

To examine process consistency, Shapley Additive Explanations (SHAP) feature attribution is conducted on the trained EMGO-SVR models. Figure 7 displays the mean absolute SHAP value ranking for the 22 upstream water quality variables. Influent suspended solids (SS-E), biochemical oxygen demand (DBO-E), chemical oxygen demand (DQO-E), and primary settler sedimentation rates (SED-E, SS-D) are identified as the most influential predictors, aligning with known wastewater physical-chemical dynamics.

### 4.4. Limitations and Scope Boundaries

This study recognizes four key scope boundaries and operational limitations:

1. **Data scope:** the case study evaluates a single municipal wastewater treatment facility; multi-plant generalization across diverse industrial wastewater streams warrants future investigation.
2. **Temporal non-stationarity:** while rolling-origin cross-validation captures expanding temporal trends, long-term seasonal concept drift and severe process upset dynamics require online continuous retraining mechanisms.
3. **Computational scaling:** the covariance update has $O(\mu D^{2})$ time complexity and $O(D^{2})$ memory requirements, which is efficient for standard dimensions (D≤100) but motivates diagonal or block-diagonal approximations for ultra-high-dimensional feature selection (D>1000).
4. **Measurement uncertainty:** in practical plant operations, laboratory sensor precision and analytical repeatability bands (≈±2 mg/L) should be considered when assessing the operational significance of numerical error reductions.

## 5. Conclusions

This study developed and evaluated the Enhanced Moss Growth Optimization (EMGO) algorithm to address the challenges of exploratory step regulation and elite correlation exploitation in continuous optimization problems. EMGO formalizes two core mathematical augmentations within the baseline MGO framework: a budget-adaptive jump regulation mechanism that smoothly transitions from expansive global exploration to refined local search, and a shrinkage-regularized covariance-guided sampling operator with relative eigenvalue flooring that reliably models elite correlation structures in high-dimensional spaces without numerical rank deficiency.

Comprehensive benchmark evaluations on the 29 CEC2017 test functions across 50 and 100 dimensions demonstrate that EMGO achieves highly competitive performance against established optimizers, including CMA-ES, L-SHADE, SBO, and baseline MGO. Nonparametric Friedman ranking, Holm-corrected Wilcoxon signed-rank tests, rank-biserial effect sizes, runtime-matched comparisons, and Latin-hypercube parameter sensitivity analyses confirm the robustness, computational efficiency, and stability of the proposed mechanisms.

Furthermore, EMGO was successfully applied to optimize support vector regression hyperparameters for effluent suspended solid prediction in a wastewater treatment facility under a rigorous expanding-window rolling-origin protocol. EMGO-SVR demonstrated statistically superior prediction accuracy and a lower generalization error than baseline metaheuristic-tuned SVRs and tree-based machine learning models. SHAP-based feature importance analysis validated the physical consistency of the learned model with wastewater treatment dynamics. Future research will explore diagonal and low-rank covariance approximations for ultra-high-dimensional domains and integrate online adaptive learning to manage temporal concept drift in dynamic industrial environments.

## Figures and Tables

**Table 1:** Performance summary of EMGO and comparator algorithms on 50 D and 100 D CEC2017 benchmark functions across 30 independent runs.

Algorithm	50 D			100 D		
	Rank	Best	Mean $\log_{10}$ Error	Rank	Best	Mean $\log_{10}$ Error
EMGO	2.48	7	3.48	2.83	9	3.82
L-SHADE	1.97	15	3.43	2.03	11	3.66
CMA-ES	3.24	5	3.59	2.45	9	3.73
SBO	3.38	2	3.66	3.38	0	3.90
MGO	4.72	0	3.89	4.97	0	4.23
HGS	6.24	0	4.13	6.03	0	4.45
FATA	7.41	0	4.30	7.07	0	4.58
RUN	7.59	0	4.35	8.48	0	4.78
TKOA	8.83	0	4.56	8.79	0	4.89
SA	9.66	0	4.65	9.69	0	5.03
PSO	10.48	0	4.84	10.28	0	5.17

**Table 2:** Wilcoxon signed-rank test results, Holm-adjusted *p*-values, and rank-biserial effect sizes between EMGO and comparator algorithms.

Comparator	50 D			100 D		
	p	$p_{\mathrm{Holm}}$	Effect	p	$p_{\mathrm{Holm}}$	Effect
L-SHADE	$3.36\times 10^{-1}$	$3.36\times 10^{-1}$	−0.21	$1.06\times 10^{-2}$	$3.17\times 10^{-2}$	−0.54
CMA-ES	$2.74\times 10^{-2}$	$5.48\times 10^{-2}$	0.47	$3.93\times 10^{-1}$	$3.93\times 10^{-1}$	−0.19
SBO	$4.79\times 10^{-3}$	$1.44\times 10^{-2}$	0.59	$1.76\times 10^{-1}$	$3.51\times 10^{-1}$	0.29
MGO	$1.19\times 10^{-5}$	$4.77\times 10^{-5}$	0.85	$1.38\times 10^{-5}$	$5.52\times 10^{-5}$	0.84
HGS	$5.22\times 10^{-8}$	$2.61\times 10^{-7}$	0.97	$2.61\times 10^{-7}$	$1.30\times 10^{-6}$	0.94
FATA	$3.73\times 10^{-9}$	$3.73\times 10^{-8}$	1.00	$3.73\times 10^{-9}$	$3.73\times 10^{-8}$	1.00
RUN	$1.86\times 10^{-8}$	$1.12\times 10^{-7}$	0.99	$7.45\times 10^{-9}$	$4.47\times 10^{-8}$	1.00
TKOA	$3.73\times 10^{-9}$	$3.73\times 10^{-8}$	1.00	$3.73\times 10^{-9}$	$3.73\times 10^{-8}$	1.00
SA	$3.73\times 10^{-9}$	$3.73\times 10^{-8}$	1.00	$3.73\times 10^{-9}$	$3.73\times 10^{-8}$	1.00
PSO	$3.73\times 10^{-9}$	$3.73\times 10^{-8}$	1.00	$3.73\times 10^{-9}$	$3.73\times 10^{-8}$	1.00

*p* denotes the raw asymptotic *p*-value of the two-sided Wilcoxon signed-rank test; $p_{Holm}$ denotes the *p*-value adjusted by Holm’s step-down procedure; Effect denotes the rank-biserial correlation effect size ($r_{rb}$).

**Table 3:** Mean wall-clock execution time in seconds over 30 independent runs on 30 D, 50 D, and 100 D benchmark functions.

Algorithm	30 D	50 D	100 D
EMGO	12.6 ± 0.6	50.2 ± 2.6	334.3 ± 20.9
L-SHADE	8.9 ± 0.5	19.8 ± 0.9	57.5 ± 2.7
CMA-ES	15.5 ± 0.9	63.8 ± 3.4	439.0 ± 27.5
SBO	6.3 ± 0.3	14.1 ± 0.7	40.5 ± 2.3
MGO	4.2 ± 0.3	9.5 ± 0.4	27.9 ± 1.5
HGS	5.6 ± 0.3	12.2 ± 0.7	35.3 ± 1.4
FATA	5.8 ± 0.3	13.1 ± 0.6	39.1 ± 2.0
RUN	7.2 ± 0.4	15.4 ± 0.9	45.5 ± 2.0
TKOA	5.9 ± 0.4	12.9 ± 0.7	37.7 ± 2.0
SA	4.9 ± 0.3	10.9 ± 0.5	31.3 ± 1.6
PSO	4.1 ± 0.2	9.1 ± 0.4	26.3 ± 1.6

**Table 4:** Runtime-matched rank comparisons under fixed wall-clock time budgets.

Algorithm	50 D/30 s	50 D/60 s	100 D/120 s	100 D/300 s
EMGO	5.0	2.0	7.0	3.0
L-SHADE	1.0	1.0	1.0	1.0
CMA-ES	8.0	4.0	6.0	5.0
SBO	2.0	3.0	2.0	2.0
MGO	3.0	5.0	3.0	4.0
HGS	4.0	6.0	4.0	6.0
FATA	6.0	7.0	5.0	7.0
RUN	7.0	8.0	8.0	8.0
TKOA	9.0	9.0	9.0	9.0
SA	10.0	10.0	10.0	10.0
PSO	11.0	11.0	11.0	11.0

**Table 5:** Ten best-performing configurations in the 60-point Latin-hypercube parameter design.

Design	μ	$\sigma_{t}$	$c_{c}$	Response
51	10	0.127	0.240	1.686
41	8	0.138	0.080	1.687
20	6	0.054	0.209	1.725
43	7	0.110	0.104	1.771
33	9	0.269	0.188	1.775
9	6	0.106	0.320	1.807
55	9	0.208	0.362	1.832
5	6	0.121	0.144	1.833
15	9	0.071	0.494	1.845
52	10	0.075	0.094	1.846

**Table 6:** Effluent suspended solid prediction performance across ten rolling-origin folds and five seeds per fold.

Method	RMSE	MAE	MAPE (%)	$R^{2}$
EMGO-SVR	5.58 ± 0.64	3.97 ± 0.46	9.07 ± 1.04	0.889 ± 0.028
BO-GP-SVR	6.02 ± 0.63	4.26 ± 0.44	9.73 ± 1.05	0.874 ± 0.029
XGBoost	6.08 ± 0.59	4.30 ± 0.44	9.85 ± 0.95	0.871 ± 0.026
LightGBM	6.06 ± 0.61	4.28 ± 0.41	9.85 ± 1.03	0.872 ± 0.029
SBO-SVR	6.04 ± 0.72	4.26 ± 0.53	9.82 ± 1.20	0.872 ± 0.032
MGO-SVR	6.22 ± 0.63	4.41 ± 0.45	10.12 ± 1.03	0.865 ± 0.027
RandomForest	6.25 ± 0.71	4.44 ± 0.51	10.13 ± 1.15	0.864 ± 0.033
RandomSearch	6.40 ± 0.60	4.53 ± 0.42	10.40 ± 1.01	0.856 ± 0.027
SA-SVR	6.45 ± 0.67	4.58 ± 0.49	10.50 ± 1.15	0.854 ± 0.034
PSO-SVR	6.99 ± 0.59	4.96 ± 0.43	11.29 ± 1.03	0.832 ± 0.032
Ridge	7.72 ± 0.62	5.46 ± 0.46	12.46 ± 1.04	0.792 ± 0.035
DefaultSVR	8.82 ± 0.67	6.24 ± 0.49	14.27 ± 1.16	0.729 ± 0.045

**Table 7:** Paired outer-fold RMSE analysis between EMGO-SVR and comparator models.

Comparator	ΔRMSE	95% CI	$p_{\mathrm{Holm}}$	Effect	W/T/L
BO-GP-SVR	−0.43	[−0.60, −0.27]	$2.15\times 10^{-2}$	−0.96	9/0/1
DefaultSVR	−3.23	[−3.35, −3.11]	$2.15\times 10^{-2}$	−1.00	10/0/0
LightGBM	−0.47	[−0.69, −0.25]	$2.15\times 10^{-2}$	−0.89	9/0/1
MGO-SVR	−0.64	[−0.79, −0.47]	$2.15\times 10^{-2}$	−1.00	10/0/0
PSO-SVR	−1.41	[−1.55, −1.26]	$2.15\times 10^{-2}$	−1.00	10/0/0
RandomForest	−0.67	[−0.79, −0.53]	$2.15\times 10^{-2}$	−1.00	10/0/0
RandomSearch	−0.82	[−0.97, −0.66]	$2.15\times 10^{-2}$	−1.00	10/0/0
Ridge	−2.13	[−2.29, −1.98]	$2.15\times 10^{-2}$	−1.00	10/0/0
SA-SVR	−0.87	[−1.04, −0.69]	$2.15\times 10^{-2}$	−1.00	10/0/0
SBO-SVR	−0.46	[ −0.63, −0.30 ]	$2.15\times 10^{-2}$	−1.00	10/0/0
XGBoost	−0.50	[ −0.68, −0.33 ]	$2.15\times 10^{-2}$	−1.00	10/0/0

## Data Availability

The UCI Water Treatment Plant dataset is openly available from the UC Irvine Machine Learning Repository at https://archive.ics.uci.edu/dataset/107/water+treatment+plant (accessed on 15 July 2026). The experimental scripts, benchmark logs, and model evaluation routines are archived in Appendix B.

## Abbreviations

The following abbreviations are used in this manuscript:

EMGO	Enhanced Moss Growth Optimization
MGO	Moss Growth Optimization
CMA-ES	Covariance Matrix Adaptation Evolution Strategy
L-SHADE	Linear Population Size Reduction Success-History Based Adaptive Differential Evolution
SVR	Support Vector Regression
SHAP	Shapley Additive Explanations
LHS	Latin-Hypercube Sampling
IQR	Interquartile Range
CD	Critical Difference
SS	Suspended Solids
DBO	Biochemical Oxygen Demand (Spanish: Demanda Bioquímica de Oxígeno)
DQO	Chemical Oxygen Demand (Spanish: Demanda Química de Oxígeno)
SED	Sedimentation
RMSE	Root-Mean-Square Error
MAE	Mean Absolute Error
MAPE	Mean Absolute Percentage Error

## Appendix A. Supplementary Benchmark Configurations

**Table A1:** Algorithm configurations and hyperparameter settings for EMGO and comparator algorithms.

Algorithm	Algorithmic Configuration and Parameters
EMGO	N=30, $JF_{\max}=3$, μ=8, $c_{c}=0.20$, $\sigma_{t}=0.10$, α=0.10, $\tau =10^{-8}$, $R_{\max}=10$
MGO	N=30, $d_{1}=0.20$, $d_{2}=0.50$, propagation probability 0.80, $R_{\max}=10$
CMA-ES	λ=30, μ=15, standard rank-μ covariance and cumulative step-size adaptation
L-SHADE	$N_{init}=18D$, $N_{min}=4$, H=5, $p_{min}=0.05$, $r_{arc}=2.6$
SBO/HGS/FATA/RUN/TKOA	Published default parameter controllers with N=30 and common evaluation budget
PSO	N=30, $c_{1}=c_{2}=2.0$, inertia weight w:0.9→0.4
SA	Initial temperature $T_{0}=100$, geometric cooling coefficient γ=0.95

## Appendix B. Supplementary Experimental Data Package

The supplementary experimental package contains complete run-level CEC2017 benchmark evaluations, execution time logs, runtime-matched summaries, 60-point Latin-hypercube parameter exploration data, condition-number trajectories, rolling-origin fold/seed predictions, and paired statistical test results. All data integrity, rank-sum, best-count, and fold-count validations are archived at https://archive.ics.uci.edu/dataset/107/water+treatment+plant (accessed on 15 July 2026).
